# *In vitro* **antimicrobial activity of** *Bixa orellana L* **. Leaves extract against anaerobic bacteria associated to bacterial vaginosis and** *Lactobacillus* spp.

**DOI:** 10.17843/rpmesp.2022.394.11978

**Published:** 2022-12-27

**Authors:** Jenny Marcas, Liz Romero, Oswaldo Tipiani, Steev Loyola, Jesús Tamariz

**Affiliations:** 1 Antimicrobial Resistance and Immunopathology Laboratory, Universidad Peruana Cayetano Heredia, Lima, Peru Universidad Peruana Cayetano Heredia Antimicrobial Resistance and Immunopathology Laboratory Universidad Peruana Cayetano Heredia Lima Peru; 2 Hospital Municipal de Surco, Lima, Peru Hospital Municipal de Surco Lima Peru; 3 Medical school, Universidad Peruana Cayetano Heredia, Lima, Peru Universidad Peruana Cayetano Heredia Medical school Universidad Peruana Cayetano Heredia Lima Peru

**Keywords:** Bixa orellana, Vaginosis, bacterial, Plant Extracts, *in vitro* Techniques, Gardnerella vaginalis, Lactobacillus

## Abstract

**Objective.:**

To describe the *in vitro* antimicrobial activity of the methanolic extract of *Bixa orellana L.* leaves against anaerobic bacteria associated to bacterial vaginosis and *Lactobacillus* spp.

**Materials and methods.:**

Eight ATCC reference strains; *Gardnerella vaginalis, Prevotella bivia*, *Peptococcus niger*, *Peptostreptococcus anaerobius*, *Mobiluncus curtisii*, *Atopobium vaginae*, *Veillonella parvula*, and *Lactobacillus crispatus*, and twenty-two clinical isolates; eleven *Gardnerella vaginalis* and eleven *Lactobacillus* strains, were included in the study*.* The antimicrobial susceptibility was determined by the agar diffusion method. The minimum inhibitory concentration (MIC) and minimum bactericidal concentration (MBC) were determined by using agar dilution and a modified dilution plating method, respectively.

**Results.:**

All ATCC reference strains showed high levels of susceptibility to the extract, except *P. vibia*, *V. parvula* and *L. crispatus*. Interestingly, all *G. vaginalis* clinical isolates and the *G. vaginalis* ATTC strain were the most susceptible to the extract, given their low MIC (1.0 - 2.0 mg/mL) and MBC (1.0 - 4.0 mg/mL) values, whereas, the *Lactobacillus spp.* clinical isolates and the *L. crispatus* ATCC strain were the least susceptible bacteria given their high MIC (32.0 mg/mL) and MBC (≥ 32.0 mg/mL) values.

**Conclusions.:**

*In vitro* experiments suggest that the extract possesses selective antimicrobial properties given its high activity against bacterial vaginosis-associated anaerobic bacteria and low activity against *Lactobacillus* species.

## INTRODUCTION

Vaginal infections cause more than 10 million healthcare visits annually, mainly due to bacterial vaginosis, candidiasis, and trichomoniasis ^1^^,^^2^. Bacterial vaginosis (BV) is the most common vaginal infection among reproductive-aged women, and it is characterized by the reduction or replacement of lactobacilli and an increase of opportunistic anaerobic bacteria ^(3-5)^. Members of the genus *Lactobacillus*, such as *L. crispatus, L. jensenii and L. iners*, are distinctive markers of a healthy vaginal microbiome, while *Gardnerella vaginalis, Prevotella, Atopobium, Mobiluncus* and *Peptostreptococcus* are frequent BV-associated anaerobic bacteria ^3^^,^^6^.

Oral metronidazole or vaginal clindamycin are routinely used as first-line treatment options for BV, however, several pathogenic bacteria, such as *Mobiluncus* and *Atopobium* species, are commonly resistant to those antimicrobials ^(3,7-10)^. The intermittent and long-term use of metronidazole or clindamycin, and infections with resistant bacteria, often result in high recurrence rates of BV within the first 12 months of treatment ^(10-12)^. Additionally, the increase of antimicrobial resistance in anaerobic bacteria after treatment has been also reported ^2^^,^^7^^,^^8^^,^^13^.

Despite the increased antimicrobial resistance and high relapse rates, the development of new drugs has been scarce, so reliance on available treatments remains necessary ^11^^,^^14^. Plant-derived compounds represent a potential source of therapeutic options for a variety of bacterial infections due to their reduced cost and toxicity, as well as their reduced risk of side effects ^15^^,^^16^. *Bixa orellana L*. is known in traditional medicine for its pharmacological properties, antimicrobial activity, and reduced toxicity ^(17-19)^. Thus, *B. orellana* constitutes a source of molecules with promising therapeutic potential that could substitute synthetic drugs used to treat BV given its antimicrobial effectiveness ^19^. The aim of the study was to evaluate the *in vitro* antimicrobial activity of *B. orellana* against BV-associated anaerobic bacteria and *Lactobacillus* spp. strains.

**KEY MESSAGES**

Motivation for the study: bacterial vaginosis is a bacterial infection that frequently affects women of reproductive age. The treatment is based on synthetic antimicrobials. *Bixa orellana L.* possesses antimicrobial properties and could represent a potential non-synthetic therapeutic alternative.

Main findings: *in vitro* results suggest that, methanolic extract of *Bixa orellana L.* leaves possesses potential antimicrobial properties against bacteria associated to bacterial vaginosis.

Implications: to identify new sources with therapeutic potential, and to promote research, discovery, and characterization of non-synthetic antimicrobials.

## MATERIALS AND METHODS

### Study design and Plant material

Experimental study that preliminarily assessed the *in vitro* antimicrobial activity of the methanolic extract of *B. orellana*. The leaves of *B. orellana* were collected in the Chanchamayo province (11.1215° S, 75.3587° W) of the Junín region of Peru, between December 2018 and January 2019. During the collection period, the daily average temperature in Junín was 10,0*°*C *(range:* 4.0°C - 15.0°C), and the daily average rainfall was 23.7 mm, with up to 100.0% of relative humidity. Collection was carried out by trained field personnel between 10:00 and 14:00. The identification of *B. orellana* was based on its botanical characteristics and on a simplified phytochemical analysis performed by botanical experts from the Museo de Historia Natural of the Universidad Nacional Mayor de San Marcos and from the Universidad Peruana Cayetano Heredia (UPCH), respectively. A voucher specimen was deposited at UPCH (CDCJ2018).

### Extract preparation

The leaves of the *B. orellana* were cleaned, oven-dried during four days at 40°C, and then ground. A liter of methanol (Germany, Darmstadt, Merck; catalog number: 106009) was added to 200 g of the product, then the mixture was macerated for seven days at room temperature in an amber glass container. The container was shaken by hand once for five minutes every day. Then, the mixture was filtered using Whatman N°2 papers, and then oven-dried at 40°C until the methanol evaporated resulting in a pasty-like extract. Finally, the extract was diluted in 40% dimethyl sulfoxide (DMSO) (Germany, Darmstadt, Merck; catalog number: D9170) to yield a final concentration of 0.5 mg/µL. The extract used in the experiments was liquid and had a dark green-brown color.

### Collection, cultivation, and identification of microorganisms

The antimicrobial activity of the extract was assessed against two bacterial groups; American Type Culture Collection (ATCC) reference strains, and clinical isolates. 

The first group consisted of eight ATCC strains. *Prevotella bivia* (ATCC 29303), *Peptococcus niger* (ATCC 27731), *Peptostreptococcus anaerobius* (ATCC 27337), *Mobiluncus curtisii* (ATCC 43063), *Atopobium vaginae* (ATCC BAA-55) and *Veillonella parvula* (ATCC 10790) were anaerobically recovered using GasPak jars (USA, Maryland, Becton Dickinson; catalog number: 260626), while *Lactobacillus crispatus* (ATCC 33197) and *Gardnerella vaginalis* (ATCC 14018) were recovered in a microaerophilic environment (USA, Massachusetts, Thermo Fisher; catalog number: AN0025A). Bacterial cultures were performed on Columbia blood agar (Germany, Darmstadt, Merck; catalog number: 27688), Human blood tween (HBT) agar, and Man-Rogosa-Sharpe (MRS) agar (Germany, Darmstadt, Merck; catalog number: 1106600500) at 37°C as described in previous studies ^20^^-^^22^. *L. crispatus* (ATCC 33197) was included as a reference for the *Lactobacillus* complex that is found in the healthy human vagina, since this study preliminarily assessed the antimicrobial activity of the extract, while the other ATCC strains were used as references of BV-associated anaerobic pathogens ^3^^,^^6^.

The second group consisted of twenty-two clinical isolates; eleven *G. vaginalis* and eleven *Lactobacillus* spp. These bacteria were isolated between September and December 2019 from samples obtained from written-informed adult women under an Institutional Review Board-approved research project (code: 104179) at the UPCH and Hospital Municipal de Surco in Lima, Peru. Vaginal discharge samples were collected and transported at 4°C from the Hospital to UPCH, and processed within two hours of collection. The diagnosis of BV was performed using the Amsel criteria ^23^, and positive samples with *Lactobacillus* were cultivated on MRS agar for the selective growth of lactobacilli ^21^. BV-positive samples were also cultured on HBT agar for the isolation of *G. vaginalis*^22^. Plates were then incubated in GasPak anaerobic jars using anaerobic or microaerophilic generators (USA, Massachusetts, Thermo Fisher; catalog number: AN0025A) for 72 hours. *G. vaginalis* and *Lactobacillus spp.* were identified using the Neisseria Haemophilus (USA, North Carolina, Biomérieux; catalog number: 21346), and Anaerobes and Corynebacteria (USA, North Carolina, Biomérieux; catalog number: 21347) identification cards in the Vitek2 system, respectively. 

### Antimicrobial susceptibility testing

*Disk diffusion agar method*

**Figure 1:**
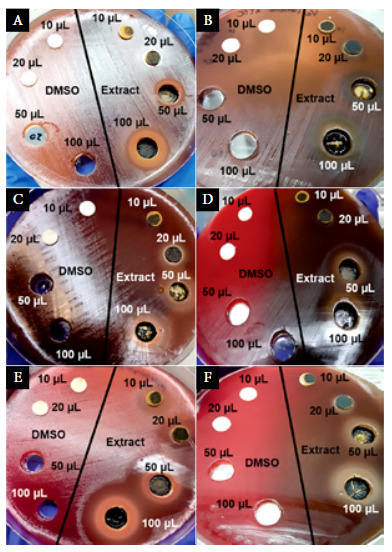
Antimicrobial susceptibility testing using 40% DMSO and *B. orellana* extract against anaerobic bacteria; a) *Prevotella bivia* ATCC 29303, b) *Veillonella parvula* ATCC 10790, c) *Atopobium vaginae* ATCC BAA-55, d) *Mobiluncus curtisii* ATCC 43063, e) *Peptoestreptococcus anaerobius* ATCC 27337, and f) *Peptococcus niger* ATCC 27731. *Sterile paper* disks and wells on the left side of the plates were used for testing the activity of 40% DMSO, and those on the right side were used for testing the extract.

Bacteria were suspended in 0.85% saline (8.5 g/L NaCl) and turbidity was adjusted to the McFarland standard No. 0.5. Using a sterile swab, the inoculum of *Lactobacillus* was spread on MRS agar, and other bacterial species was spread on Columbia blood agar. Subsequently, four wells were made on the agar with the back of a sterile 1 mL pipette tip. In addition, four sterile 6-mm paper disks (UK, Hampshire, Oxoid; catalog number: CT0998B) were placed on the agar (Figure 1). Two wells and two paper disks were used to evaluate 40% DMSO as a bacterial growth inhibitor, and the other wells and disks were used to test the antimicrobial activity of the extract.

The activity of 40% DMSO (Figure 1, on the left side of the plates) was assessed at different volumes using 1:1 dilution with saline solution; disks were soaked with 10 and 20 µL, and wells were filled with 50 and 100 µL. Correspondingly, the antimicrobial activity of the extract was assessed using the same volumes and procedures (Figure 1, on the right side of the plates). The plates were incubated at 37°C, and after three days the inhibition zones were recorded and interpreted according to the Duraffourd scale ^24^.

*Minimum Inhibitory Concentration (MIC)*

The extract at concentrations of 1, 2, 4, 8, 16, and 32 mg/mL was added to the Columbia agar supplemented with 5% sheep blood, as previously described ^25^. Ten microliters of a bacterial suspension with turbidity equivalent to the McFarland standard N°. 0.5 were inoculated onto each plate. The plates were incubated in an anaerobic or microaerophilic environment at 37°C, depending on the bacterial requirements for growth, and MIC was defined as the lowest concentration of the extract at which no bacterial growth was observed ^25^. Three technical replicates were performed for each assessment. Bacterial strains were inoculated on Columbia agar supplemented with 5% sheep blood as positive controls.

*Minimum Bactericidal Concentration (MBC)*

A previously described method, with some modifications, was used as reference because MBC could not be determined with a conventional liquid method since the extract had a dark color^26^. The inoculated region with no bacterial growth was scraped from plates used for MIC determination with a sterile loop and then gently spread onto a new plate with non-selective agar. Based on bacterial requirements for growth, plates were incubated in either anaerobic or microaerophilic conditions at 37°C for 72 hours. The MBC was defined as the lowest extract concentration at which no bacterial growth was observed. Three technical replicates were performed for each MBC determination.

*Statistical Analysis*

Technical triplicates had no significant variation; therefore, data were reported using averages ± standard deviations (SD). The MIC_50_, MIC_90_, MBC_50_ and MBC_90_ were estimated for the group of clinical isolates. Lastly, the MIC and MBC differences between clinical isolates of *G. vaginalis* and *Lactobacillus* were evaluated using the Mann-Whitney U test. Data analysis was performed in Stata v15 (StataCorp., College Station, TX, USA) considering a value of p<0.050 as significant.

## RESULTS

The 40% DMSO, regardless of the volume used, had no bactericidal effect neither inhibited the bacterial growth of the ATCC reference strains used. Figure 1 (left side of the plates) shows the results for some ATCC reference strains.

*Antimicrobial activity against ATCC reference strains*

The inhibition zone was directly related to the volume of extract used (Table 1). The extract exhibited high antimicrobial activity against all ATCC reference strains, except for *L. crispatus* ATCC 33197, *P. bivia* ATCC 29303, and *V. parvula* ATCC 10790 (Table 1). Interestingly, the growth of *L. crispatus* ATCC 33197 was not inhibited regardless of the volume used, and the growth of *V. parvula* ATCC 10790 was only inhibited by 100 µL of the extract.

**Table 1:** *In vitro* activity of *B. orellana L*. extract against ATCC reference strains.

ATCC Bacteria	Inhibition zone (mm)				MIC (mg/mL)	MBC (mg/mL)
	10 µl ^a^	20µl ^a^	50 µl ^a^	100µl ^a^		
*G. vaginalis* (ATCC 14018)	14.0 ± 1.0	20.3 ± 1.5	24.7 ± 0.6	30.3 ± 0.6	1.0	4.0
*L. crispatus* (ATCC 33197)	0.0 ± 0.0	0.0 ± 0.0	0.0 ± 0.0	0.0 ± 0.0	32.0	32.0
*A. vaginae* (ATCC BAA-55)	0.0 ± 0.0	12.0 ± 1.0	14.0 ± 1.0	18.3 ± 0.6	4.0	4.0
*M. curtisii* (ATCC 43063)	0.0 ± 0.0	12.3 ± 1.2	18.3 ± 0.6	20.0 ± 1.0	4.0	8.0
*P. niger* (ATCC 27731)	13.3 ± 0.6	15.0 ± 1.0	22.3 ± 0.6	25.0 ± 0.0	8.0	8.0
*P. anaerobius* (ATCC 27337)	13.7 ± 1.5	18.7 ± 0.6	25.7 ± 1.2	28.3 ± 0.6	4.0	8.0
*P. bivia* (ATCC 29303)	0.0 ± 0.0	10.3 ± 0.6	12.0 ± 1.0	18.7 ± 1.2	32.0	32.0
*V. parvula* (ATCC 10790)	0.0 ± 0.0	0.0 ± 0.0	0.0 ± 0.0	12.3 ± 1.5	32.0	>32.0

a Mean ± standard deviation

The MIC and MBC for ATCC stains are reported in Table 1. *G. vaginalis* ATCC 14018 was the most susceptible bacteria, with MIC and MBC of 1.0 mg/mL and 4.0 mg/mL, respectively. *P. bivia* ATCC 29303, *V. parvula* ATCC 10790 and *L. crispatus* ATCC 33197 were the most resistant bacteria, displaying higher MIC and MBC values compared to other ATCC strains.

*Antimicrobial activity against clinical isolates*

Among *G. vaginalis* clinical isolates, the most susceptible was strain M10 (MIC and MBC: 1.0 mg/mL), followed by strains MD (MIC: 1.0 mg/mL; MBC: 4.0 mg/mL) and M23 (MIC and MBC: 2.0 mg/mL) while the other eight isolates displayed MIC and MBC values of 2.0 mg/mL and 4.0 mg/mL, respectively (Table 2). Overall, the MIC_50_ and MIC_90_ were 2 mg/mL, and the MBC_50_ and MBC_90_ were 4.0 mg/mL.

*Lactobacillus* spp. isolates grew abundantly over the range of 1.0 to 16.0 mg/mL of the extract. However, there was no evidence of bacterial growth at 32.0 mg/mL. Consequently, the MIC value for all clinical isolates of *Lactobacillus* spp*.* was 32.0 mg/mL (Table 2), and the MIC_50_ and MIC_90_ were 32.0 mg/mL. Regarding the MBC, 54.5% (6/11) of the *Lactobacillus* spp. isolates (M7, M24, M25, M33, MA and MB; Table 2) showed a value of 32.0 mg/mL, while other clinical isolates showed values above 32.0 mg/mL. The MBC_50_ and the MBC_90_ for *Lactobacillus* spp. were 32.0 mg/mL and >32.0 mg/mL, respectively.

**Table 2:** Minimum inhibitory (MIC) and minimum bactericidal concentration (MBC) in ATCC reference strains and clinical isolates of *G. vaginalis* and *Lactobacillus* spp.

Bacteria^a^		MIC	MBC
*G. vaginalis*			
	M10 (n=1)	1.0 mg/mL	1.0 mg/mL
	ATCC 14018 and MD (n=2)	1.0 mg/mL	4.0 mg/mL
	M23 (n=1)	2.0 mg/mL	2.0 mg/mL
	MG, M5, M8, M9, M26, M29, M30 and M31 (n=8)	2.0 mg/mL	4.0 mg/mL
*Lactobacillus* spp.			
	ATCC 33197, M7, M24, M25, M33, MA and MB (n=7)	32.0 mg/mL	32.0 mg/mL
	M1, M3, M4, M6 and M27 (n=5)	32.0 mg/mL	>32.0 mg/mL

a Clinical isolates were coded as: M#, MD, MG, MA, or MB

The extract exhibited a significant and differential antimicrobial activity between *G. vaginalis* and *Lactobacillus* spp. strains (p<0.001). Specifically, *G. vaginalis* isolates were inhibited at low concentrations of the extract (MIC: 2.0 mg/mL, MBC: 4.0 mg/mL), while *Lactobacillus* spp. isolates were inhibited at higher concentrations (MIC: 32.0 mg/mL, MBC: 3.0 mg/mL).

## DISCUSSION

In recent years, the use of medicinal plants or herbs has been promoted by scientific research due to their pharmacological activity, low toxicity, and inexpensive accessibility ^15^^,^^16^^,^^19^^,^^25^. Plants possess and produce a wide variety of secondary metabolites such as tannins, terpenoids, flavonoids, glycosides, saponins, anthranoids, quinones and coumarins, to which antimicrobial properties have been attributed ^27^. Several studies suggest that *B. orellana* possesses various properties, including antimicrobial, antifungal, antioxidant, anti-inflammatory, and analgesic activity ^(19, 28)^. The antimicrobial activity of *B. orellana* against several microorganisms has been previously described ^19^^,^^28^, however, its antimicrobial activity on microaerophilic or anaerobic bacteria responsible for BV has been scarcely described.

Our results suggest that, in the absence of bacteriostatic or bactericidal activity of the 40% DMSO, the observed antimicrobial activity can be attributed to the extract. This finding is consistent with several other studies that suggest that *B. orellana* leaves extracts, obtained by methanol- or ethanol-based techniques, have antimicrobial activity against reference ATCC strains and clinical isolates ^19^^,^^29^^,^^30^. Therefore, based on our results and the results reported by other studies ^19^^,^^28^^-^^30^, it is reasonable to consider that the evaluated extract possesses antimicrobial activity.

BV is a dysbiosis characterized by drastic changes in the biota of the vaginal tract that result in a replacement of the lactobacillus-predominant vaginal flora by anaerobic bacteria ^3^^,^^6^. *G. vaginalis* is one of the most frequent anaerobic bacteria causing BV, which symbiotically can form a polymicrobial biofilm with several BV-associated anaerobic bacteria such as *A. vaginae* and *Prevotella* spp ^2^^,^^6^^,^^31^. Other anaerobic bacteria commonly detected in women with BV are *Peptoestreptococcus* and *Mobiluncus*^6^^,^^32^^,^^33^. Based on the observed low MIC and MBC values, it can be inferred that the evaluated extract has a high antimicrobial activity against most of the ATCC reference strains used here and also against clinical isolates of *G. vaginalis*. However, it is important to note that *P. bivia* and *V. parvula*, both ATCC strains, displayed low susceptibility to the extract. To the best of our knowledge, the *in vitro* activity of the *B. orellana* extract against *G. vaginalis* and other anaerobic pathogens has been scarcely described. Further studies are needed to validate our findings in a wide variety of clinical isolates. 

Vaginal lactobacilli regulate pH and protect the mucosa against the establishment of pathogenic microorganisms ^3^^,^^33^. In this study, *L. crispatus* ATCC 33197 and clinical isolates of *Lactobacillus* spp. were used as references of the *Lactobacillus* complex found in the healthy human vagina ^(^^6^^,^^32^. Interestingly, the extract inhibited the growth of lactobacilli at concentrations of 32.0 mg/mL. However, our results differ from those described elsewhere. Galindo-Cuspinera *et al*. ^34^ suggested that the extract obtained from fruits and seeds of *B. orellana* exhibited limited antimicrobial activity against *L. lactis* ATCC 11454 and *L. casei* ATCC 39539, and no activity against *L. plantarum* ATCC 700210. Similarly, Ogunshe *et al*. ^35^ suggested that *B. orellana* had limited *in vitro* activity against various vaginal *Lactobacillus* spp. strains. Overall, these discrepant results could be explained by; *i*) the use of different extraction methods (i.e., type of extract and solvent) resulting in variable concentration of phytochemicals, *ii*) the use of different parts of *B. orellana* (i.e., leaves, seeds, pods or fruits) for extract preparation, *iii*) the geographical location and exposure to climatic and environmental factors affecting *B. orellana*, and *iv*) by intrinsic differences between *Lactobacillus* spp. strains used in other studies ^(^^19^.

Overall, our findings are consistent with several other studies suggesting that *B. orellana* extract possesses antimicrobial activity ^(17-19,28,30)^. An important major strength of this *in vitro* study is the evaluation of the antimicrobial activity of the extract against anaerobic bacteria representative of those causing BV, and against representative bacteria of the healthy vaginal biota. Our findings may support the scale-up and innovation of research for the development of potential therapeutic options. However, it is also important to consider the limitations of this *in vitro* study. Multiple phytochemicals are found in *B. orellana*, including flavonoids, polyphenols, tannins, quinones, terpenoids and alkaloids, which exert antimicrobial activity through complex processes ^19^. In this preliminary study we have not characterized the phytochemical compounds or their concentrations; therefore, we could not establish nor isolate the individual antimicrobial activity of each component of the extract against the studied bacterial isolates. Furthermore, it is reasonable to assume that the antimicrobial activity described here may not be extrapolated to all *B. orellana* species given possible intrinsic differences among them and differences in plant cultivation. 

In summary, our *in vitro* experiments suggest that the extract of *B. orellana* could be a source of antimicrobial compound or compounds with high selective antimicrobial activity against most BV-associated anaerobic bacteria, and with reduced activity against protective bacteria found in the healthy vaginal mucosa. Further research is needed to identify the compound or compounds with antimicrobial activity, in order to validate the preliminary differential activity described in our study by using a more rigorous and complex study design.
