# Supplementary material for: In vitro antimicrobial activity of Bixa orellana L . Leaves extract against anaerobic bacteria associated to bacterial vaginosis and Lactobacillus spp
Source: Rev Peru Med Exp Salud Publica. 2022 Dec 27;39(4):408–14. doi: 10.17843/rpmesp.2022.394.11978 (PMC11397780; doi:10.17843/rpmesp.2022.394.11978)
Supplement: Translation

# Translation: Actividad antimicrobiana *in vitro *del extracto de hojas de *Bixa orellana L. c*ontra bacterias anaerobias asociadas a la vaginosis bacteriana y *Lactobacillus *spp.

**Authors:** Jenny Marcas, Liz Romero, Oswaldo Tipiani, Steev Loyola, Jesús Tamariz

**Affiliations:** 1 Laboratorio de Resistencia Antimicrobiana e Inmunopatología, Universidad Peruana Cayetano Heredia, Lima, Perú; 2 Hospital Municipal de Surco, Lima, Perú; 3 Facultad de Medicina, Universidad Peruana Cayetano Heredia, Lima, Perú

**Keywords:** *Bixa orellana*, vaginosis bacteriana, Técnicas *in vitro*, *Gardnerella vaginalis*, *Lactobacillus*

## Abstract

**Objetivos:**

. Describir la actividad antimicrobiana *in vitro* del extracto metanólico de las hojas de *Bixa orellana L.* contra las bacterias anaerobias asociadas a la vaginosis bacteriana y *Lactobacillus* spp.

**Materiales y métodos:**

. Se incluyeron en el estudio ocho cepas de referencia ATCC; *Gardnerella vaginalis*, *Prevotella bivia*, *Peptococcus niger*, *Peptostreptococcus anaerobius*, *Mobiluncus curtisii*, *Atopobium vaginae*, *Veillonella parvula* y *Lactobacillus crispatus*, y 22 aislamientos clínicos; once aislados de *Gardnerella vaginalis* y once aislados de *Lactobacillus*. La susceptibilidad antimicrobiana se determinó mediante el método de difusión en agar. La concentración mínima inhibitoria (CMI) y la concentración bactericida mínima (CBM) fueron determinadas utilizando el método de dilución en agar y un método de dilución modificado, respectivamente.

**Resultados:**

. Todas las cepas de referencia ATCC tuvieron un alto nivel de susceptibilidad al extracto, con excepción de *P. vibia*, *V. parvula* y *L. crispatus*. Interesantemente, los aislamientos clínicos de *G. vaginalis* y la cepa ATCC de *G. vaginalis* fueron los más susceptibles al extracto dados los bajos valores de CMI (1,0 - 2,0 mg/mL) y CBM (1,0 - 4,0 mg/mL), mientras que, los aislamientos clínicos de *Lactobacillus* spp. y la cepa ATCC de *L. crispatus* fueron los menos susceptibles debido a los altos valores de CMI (32,0 mg/mL) y CBM (≥ 32,0 mg/mL).

**Conclusiones:**

. Los experimentos *in vitro* sugieren que el extracto posee propiedades antibacterianas selectivas dada su alta actividad contra bacterias anaerobias asociadas a vaginosis bacteriana y baja actividad contra especies de *Lactobacillus*.

## INTRODUCCIÓN

Las infecciones vaginales ocasionan más de 10 millones de visitas a los servicios de salud al año, principalmente debido a vaginosis bacteriana, candidiasis y tricomoniasis ^1^ ^,^ ^2^. La vaginosis bacteriana (VB) es la infección vaginal más frecuente entre las mujeres en edad reproductiva, y se caracteriza por la reducción o sustitución de lactobacilos y el aumento de bacterias anaerobias oportunistas ^3^ ^-^ ^5^. Los miembros del género *Lactobacillus*, como *L. crispatus*, *L. jensenii* y *L. iners*, son marcadores distintivos de un microbioma vaginal sano, mientras que *Gardnerella vaginalis*, *Prevotella*, *Atopobium*, *Mobiluncus* y *Peptostreptococcus* son bacterias anaerobias frecuentes asociadas a la VB ^3^ ^,^ ^6^.

El metronidazol oral o la clindamicina vaginal se utilizan habitualmente como opciones de tratamiento de primera línea para la VB; sin embargo, diversas bacterias patógenas, como las especies *Mobiluncus* y *Atopobium*, suelen ser resistentes a esos antimicrobianos ^3^ ^,^ ^7^ ^-^ ^10^. El uso intermitente y a largo plazo de metronidazol o clindamicina, y las infecciones por bacterias resistentes, suelen dar lugar a altas tasas de recurrencia de la VB en los primeros 12 meses de tratamiento ^10^ ^-^ ^12^. Además, también se ha notificado el aumento de la resistencia a los antimicrobianos en bacterias anaerobias después del tratamiento ^2^ ^,^ ^7^ ^,^ ^8^ ^,^ ^13^.

A pesar del aumento de la resistencia a los antimicrobianos y de las elevadas tasas de recaída, el desarrollo de nuevos fármacos ha sido escaso, por lo que sigue siendo necesario recurrir a los tratamientos disponibles ^11^ ^,^ ^14^. Los compuestos derivados de plantas representan una fuente de potenciales opciones terapéuticas para una variedad de infecciones bacterianas debido a su menor costo, toxicidad reducida, y a su menor riesgo de efectos secundarios ^15^ ^,^ ^16^. *Bixa orellana L*. es conocida en la medicina tradicional por sus propiedades farmacológicas, su actividad antimicrobiana y su reducida toxicidad ^17^ ^-^ ^19^. Por lo tanto, *B. orellana* se constituye como una fuente de fitoquímicos con prometedor potencial terapéutico que podría sustituir a los fármacos sintéticos utilizados para tratar la VB dada su eficacia antimicrobiana ^19^. El objetivo del estudio fue evaluar la actividad antimicrobiana *in vitro* de *B. orellana* contra bacterias anaerobias asociadas a la VB y cepas de *Lactobacillus* spp.

**MENSAJES CLAVE**

Motivación para realizar el estudio: la vaginosis bacteriana es una infección bacteriana que afecta de forma frecuente a las mujeres en edad reproductiva. El tratamiento se basa en antimicrobianos sintéticos. *Bixa orellana L.* posee propiedades antimicrobianas y podría representar una potencial alternativa terapéutica no sintética.

Principales hallazgos: los resultados *in vitro* sugieren que el extracto metanolico de las hojas de *Bixa orellana L.* posee potenciales propiedades antimicrobianas contra las bacterias asociadas a vaginosis bacteriana.

Implicancias: identificar nuevas fuentes con potencial terapéutico, y promover la investigación, descubrimiento y caracterización de antimicrobianos no sintéticos.

## MATERIALES Y MÉTODOS

### Diseño del estudio y material vegetal

Estudio experimental que evaluó preliminarmente la actividad antimicrobiana *in vitro* del extracto metanólico de *B. orellana*. Las hojas de *B. orellana* fueron recolectadas en la provincia de Chanchamayo (11,1215° S, 75,3587° O) de la región Junín, Perú, entre diciembre de 2018 y enero de 2019. Durante el periodo de colecta, la temperatura media diaria en Junín fue de 10,0°C (rango: 4,0°C - 15,0°C), y la precipitación media diaria fue de 23,7 mm, con hasta 100,0% de humedad relativa. La recolección fue realizada por personal de campo entrenado entre las 10:00 y las 14:00 horas. La identificación de *B. orellana* se basó en sus características botánicas ^17^ y en un análisis fitoquímico simplificado realizado por expertos botánicos del Museo de Historia Natural de la Universidad Nacional Mayor de San Marcos y de la Universidad Peruana Cayetano Heredia (UPCH), respectivamente. Se depositó un espécimen en la UPCH (CDCJ2018).

### Preparación del extracto

Se limpiaron las hojas de *B. orellana*, se secaron en un horno durante cuatro días a 40°C y se molieron. Se añadió un litro de metanol (Alemania, Darmstadt, Merck; número de catálogo: 106009) a 200 g del producto molido, y la mezcla se maceró durante siete días a temperatura ambiente en un recipiente de vidrio ámbar. Diariamente, el recipiente se agitó manualmente una vez durante cinco minutos. A continuación, la mezcla se filtró con papel Whatman N°2 y se secó en un horno a 40°C hasta que el metanol se evaporó, obteniéndose un extracto pastoso. Por último, el extracto se diluyó en dimetilsulfóxido (DMSO) al 40% (Alemania, Darmstadt, Merck; número de catálogo: D9170) para obtener una concentración final de 0,5 mg/µL. El extracto utilizado en los experimentos era líquido y tenía un color verde oscuro-marrón.

### Recolección, cultivo e identificación de microorganismos

La actividad antimicrobiana del extracto se evaluó frente a dos grupos de bacterias: cepas de referencia de la American Type Culture Collection (ATCC) y aislamientos provenientes de muestras clínicas.

El primer grupo estuvo conformado por ocho cepas ATCC. *Prevotella bivia* (ATCC 29303), *Peptococcus niger* (ATCC 27731), *Peptostreptococcus anaerobius* (ATCC 27337), *Mobiluncus curtisii* (ATCC 43063), *Atopobium vaginae* (ATCC BAA-55) y *Veillonella parvula* (ATCC 10790) se recuperaron anaeróbicamente utilizando frascos GasPak (EE.UU., Maryland, Becton Dickinson; número de catálogo: 260626), mientras que *Lactobacillus crispatus* (ATCC 33197) y *Gardnerella vaginalis* (ATCC 14018) se recuperaron en un medio microaerófilo (EE.UU., Massachusetts, Thermo Fisher; número de catálogo: AN0025A). Los cultivos bacterianos se realizaron en agar sangre Columbia (Alemania, Darmstadt, Merck; número de catálogo: 27688), agar sangre humana tween (HBT) y agar Man-Rogosa-Sharpe (MRS) (Alemania, Darmstadt, Merck; número de catálogo: 1106600500) a 37°C como se describe en otros estudios ^20^ ^-^ ^22^. Se incluyó *L. crispatus* (ATCC 33197) como referencia del complejo Lactobacillus presente en la vagina humana sana, con el propósito de evaluar la actividad del extracto frente a un marcador bacteriano distintivo de un microbioma vaginal sano; mientras que las demás cepas ATCC se utilizaron como referencias de patógenos anaerobios asociados a la VB ^3^ ^,^ ^6^.

El segundo grupo estuvo conformado por veintidós aislamientos clínicos; once de *G. vaginalis* y once de *Lactobacillus* spp. Estas bacterias se aislaron entre septiembre y diciembre de 2019 de muestras obtenidas de mujeres adultas informadas por escrito en el marco de un proyecto de investigación aprobado por la el Comité Institucional de Ética (código: 104179) de la UPCH y el Hospital Municipal de Surco en Lima, Perú. Las muestras de secreción vaginal fueron recolectadas y transportadas a 4°C desde el Hospital a la UPCH, y procesadas dentro de las dos horas siguientes a la recolección. El diagnóstico de VB se realizó utilizando los criterios de Amsel ^23^, y las muestras positivas a *Lactobacillus* se cultivaron en agar MRS para el crecimiento selectivo de lactobacilos ^21^. Las muestras positivas a VB también se cultivaron en agar HBT para el aislamiento de *G. vaginalis* ^22^. A continuación, las placas se incubaron en frascos anaeróbicos GasPak utilizando generadores anaeróbicos o microaerófilos (EE.UU., Massachusetts, Thermo Fisher; número de catálogo: AN0025A) durante 72 horas. *G. vaginalis* y *Lactobacillus* spp. se identificaron utilizando las tarjetas de identificación *Neisseria Haemophilus* (EE.UU., Carolina del Norte, Biomérieux; número de catálogo: 21346), y *Anaerobes* y *Corynebacteria* (EE.UU., Carolina del Norte, Biomérieux; número de catálogo: 21347) en el sistema Vitek2, respectivamente.

### Pruebas de susceptibilidad antimicrobiana

*Método de difusión en disco de agar*

Las bacterias se suspendieron en solución salina al 0,85% (8,5 g/L NaCl) y la turbidez se ajustó al estándar McFarland N° 0,5. Utilizando un hisopo estéril, se extendió el inóculo de *Lactobacillus* en agar MRS, y el de otras especies bacterianas en agar sangre Columbia. Posteriormente, con el dorso de una punta de pipeta estéril de 1 mL, se hicieron cuatro pocillos en el agar. Además, se colocaron sobre el agar cuatro discos estériles de papel de 6 mm (UK, Hampshire, Oxoid; número de catálogo: CT0998B) (Figura 1). Se utilizaron dos pocillos y dos discos de papel para evaluar el efecto inhibidor del crecimiento bacteriano del DMSO al 40%, y los otros pocillos y discos se utilizaron para evaluar la actividad antimicrobiana del extracto.

**Figura 1:** Pruebas de susceptibilidad antimicrobiana utilizando DMSO al 40% y extracto de *B. orellana* contra bacterias anaerobias; a) *Prevotella bivia *ATCC 29303, b) *Veillonella parvula *ATCC 10790, c) *Atopobium vaginae *ATCC BAA-55, d) *Mobiluncus curtisii *ATCC 43063, e) *Peptoestreptococcus anaerobius* ATCC 27337, y f) *Peptococcus niger *ATCC 27731. Los discos de papel estériles y pocillos en el lado izquierdo de cada placa se usaron para evaluar la actividad del DMSO al 40%, y aquellos en el lado derecho se usaron para evaluar el extracto.

La actividad del DMSO al 40% (Figura 1, en el lado izquierdo de las placas) se evaluó a diferentes volúmenes utilizando dilución 1:1 con solución salina; los discos se embebieron con 10 y 20 µl, y los pocillos se llenaron con 50 y 100 µl. Del mismo modo, se evaluó la actividad antimicrobiana del extracto utilizando los mismos volúmenes y procedimientos (Figura 1, a la derecha de las placas). Las placas se incubaron a 37°C, y al cabo de tres días se registraron las zonas de inhibición y se interpretaron según la escala de Duraffourd ^24^.

*Concentración mínima inhibitoria (CMI)*

El extracto en concentraciones de 1, 2, 4, 8, 16 y 32 mg/mL se añadió al agar Columbia suplementado con 5% de sangre de oveja, como se describió previamente ^25^. En cada placa se inocularon diez microlitros de una suspensión bacteriana con turbidez equivalente al estándar McFarland N.° 0,5. Las placas se incubaron en un ambiente anaeróbico o microaerófilo a 37°C, dependiendo de los requerimientos de crecimiento de cada especie bacteriana. La CMI se definió como la concentración más baja del extracto en la que no se observó crecimiento bacteriano ^25^. Se realizaron tres réplicas técnicas para cada evaluación. Como controles positivos se inocularon las cepas bacterianas en agar Columbia suplementado con 5% de sangre de oveja.

*Concentración bactericida mínima (CBM)*

Se utilizó como referencia un método descrito previamente con algunas modificaciones debido a que, dado el color oscuro del extracto, no fue posible determinar la CBM mediante un método convencional ^26^. A partir de las placas usadas para determinar la CMI, las regiones inoculadas sin crecimiento bacteriano evidente fueron raspadas con un asa de siembra estéril y luego se sembró en una placa con agar sin presencia de inhibidores. En función de los requerimientos bacterianos para el crecimiento, las placas se incubaron en condiciones anaeróbicas o microaerófilas a 37 °C durante 72 horas. La CBM se definió como la concentración de extracto más baja en la que no se observó crecimiento bacteriano. Se realizaron tres réplicas técnicas para cada determinación de la CBM.

### Análisis estadístico

Los triplicados técnicos no presentaron variaciones significativas; por lo tanto, los datos se reportaron utilizando medias ± desviaciones estándar (DE). Se estimaron las CMI_50_, CMI_90_, CBM_50_ y CBM_90_ para el grupo de aislados clínicos. Por último, las diferencias de CMI y CBM entre los aislados clínicos de *G. vaginalis* y *Lactobacillus* se evaluaron mediante la prueba U de Mann-Whitney. El análisis de los datos se realizó en Stata v15 (StataCorp., College Station, TX, EE.UU.) considerando significativo un valor de p<0,050.

## RESULTADOS

El DMSO al 40%, independientemente del volumen utilizado, no tuvo efecto bactericida ni bacteriostático frente a las cepas ATCC. En la Figura 1 (parte izquierda de las placas) se muestran los resultados de algunas cepas de referencia ATCC.

### Actividad antimicrobiana frente a cepas de referencia ATCC

La zona de inhibición estuvo directamente relacionada con el volumen de extracto utilizado (Tabla 1). El extracto mostró una elevada actividad antimicrobiana contra todas las cepas de referencia ATCC, excepto *L. crispatus* ATCC 33197, *P. bivia* ATCC 29303 y *V. parvula* ATCC 10790 (Tabla 1). Curiosamente, el crecimiento de *L. crispatus* ATCC 33197 no se inhibió independientemente del volumen utilizado, y el crecimiento de *V. parvula* ATCC 10790 sólo se inhibió con 100 µl del extracto.

**Tabla 1:** Actividad* in vitro* del extracto de *B. orellana L.* frente a cepas de referencia ATCC.

Bacterias ATCC	Zona de inhibición (mm)				CMI (mg/mL)	CBM (mg/mL)
	10 µl ^a^	20µl^ a^	50 µl^ a^	100µl ^a^		
*G. vaginalis *(ATCC 14018)	14,0 ± 1,0	20,3 ± 1,5	24,7 ± 0,6	30,3 ± 0,6	1,0	4,0
*L. crispatus *(ATCC 33197)	0,0 ± 0,0	0,0 ± 0,0	0,0 ± 0,0	0,0 ± 0,0	32,0	32,0
*A. vaginae *(ATCC BAA-55)	0,0 ± 0,0	12,0 ± 1,0	14,0 ± 1,0	18,3 ± 0,6	4,0	4,0
*M. curtisii *(ATCC 43063)	0,0 ± 0,0	12,3 ± 1,2	18,3 ± 0,6	20,0 ± 1,0	4,0	8,0
*P. niger *(ATCC 27731)	13,3 ± 0,6	15,0 ± 1,0	22,3 ± 0,6	25,0 ± 0,0	8,0	8,0
*P. anaerobius *(ATCC 27337)	13,7 ± 1,5	18,7 ± 0,6	25.7 ± 1,2	28.3 ± 0,6	4,0	8,0
*P. bivia *(ATCC 29303)	0,0 ± 0,0	10,3 ± 0,6	12,0 ± 1,0	18,7 ± 1,2	32,0	32,0
*V. parvula* (ATCC 10790)	0,0 ± 0,0	0,0 ± 0,0	0,0 ± 0,0	12,3 ± 1,5	32,0	>32,0

a Media ± desviación estándar

En la tabla 1 se indican la CMI y la CBM para las cepas ATCC. *G. vaginalis* ATCC 14018 fue la bacteria más susceptible, con CMI y CBM de 1,0 mg/mL y 4,0 mg/mL, respectivamente. *P. bivia* ATCC 29303, *V. parvula* ATCC 10790 y *L. crispatus* ATCC 33197 fueron las bacterias más resistentes, mostrando valores de CMI y CBM más altos en comparación con otras cepas ATCC.

### Actividad antimicrobiana frente a aislados clínicos

Entre los aislados clínicos de *G. vaginalis*, el más susceptible fue la cepa M10 (CMI y CBM: 1,0 mg/mL), seguida de las cepas MD (CMI: 1,0 mg/mL; CBM: 4,0 mg/mL) y M23 (CMI y CBM: 2,0 mg/mL), mientras que los otros ocho aislados mostraron valores de CMI y CBM de 2,0 mg/mL y 4,0 mg/mL, respectivamente (Tabla 2). En general, la CMI_50_ y la CMI_90_ fueron de 2 mg/mL, y la CBM_50_ y la CBM_90_ fueron de 4,0 mg/mL.

Los aislados de *Lactobacillus* spp. crecieron abundantemente en el intervalo de 1,0 a 16,0 mg/mL del extracto. Sin embargo, no hubo evidencia de crecimiento bacteriano a 32,0 mg/mL. En consecuencia, el valor CMI para todos los aislados clínicos de *Lactobacillus* spp. fue de 32,0 mg/mL (Tabla 2), y el CMI_50_ y CMI_90_ fueron de 32,0 mg/mL. En cuanto a la CBM, el 54,5% (6/11) de los aislados de *Lactobacillus* spp. (M7, M24, M25, M33, MA y MB; Tabla 2) mostraron un valor de 32,0 mg/mL, mientras que otros aislados clínicos mostraron valores superiores a 32,0 mg/mL. La CBM_50_ y la CBM_90_ para *Lactobacillus* spp. fueron 32,0 mg/mL y >32,0 mg/mL, respectivamente.

**Tabla 2.** Concentración mínima inhibitoria (CMI) y concentración bactericida mínima (CBM) en cepas de referencia ATCC y aislados clínicos de *G. vaginalis* y *Lactobacillus spp.*

Bacteria ^a^		CMI	MBC
*G. vaginalis *			
	M10 (n=1)	1,0 mg/mL	1,0 mg/mL
	ATCC 14018 y MD (n=2)	1,0 mg/mL	4,0 mg/mL
	M23 (n=1)	2,0 mg/mL	2,0 mg/mL
	MG, M5, M8, M9, M26, M29, M30 y M31 (n=8)	2,0 mg/mL	4,0 mg/mL
*Lactobacillus spp. *			
	ATCC 33197, M7, M24, M25, M33, MA y MB (n=7)	32,0 mg/mL	32,0 mg/mL
	M1, M3, M4, M6 y M27 (n=5)	32,0 mg/mL	>32,0 mg/mL

a Los aislados clínicos fueron codificados de la siguiente forma: M#, MD, MG, MA, y MB

El extracto mostró una actividad antimicrobiana significativa y diferencial entre las cepas de *G. vaginalis* y *Lactobacillus* spp. (p<0,001). Específicamente, los aislados de *G. vaginalis* se inhibieron a concentraciones bajas del extracto (CMI: 2,0 mg/mL, CBM: 4,0 mg/mL), mientras que los aislados de *Lactobacillus* spp. se inhibieron a concentraciones más altas (CMI: 32,0 mg/mL, CBM: 32,0 mg/mL).

## DISCUSIÓN

En los últimos años, el uso de plantas o hierbas medicinales ha sido objeto de investigación científica debido a su actividad farmacológica, baja toxicidad y accesibilidad económica ^15^ ^,^ ^16^ ^,^ ^19^ ^,^ ^25^. Las plantas poseen y producen una amplia variedad de metabolitos secundarios como taninos, terpenoides, flavonoides, glucósidos, saponinas, antranoides, quinonas y cumarinas, a los que se han atribuido propiedades antimicrobianas ^27^. Varios estudios sugieren que *B. orellana* posee diversas propiedades, como actividad antimicrobiana, antifúngica, antioxidante, antiinflamatoria y analgésica ^(19, 28)^. La actividad antimicrobiana de *B. orellana* contra varios microorganismos ha sido descrita previamente ^19^ ^,^ ^28^, sin embargo, su actividad antimicrobiana sobre bacterias microaerófilas o anaeróbicas responsables de la VB ha sido escasamente descrita.

Nuestros resultados sugieren que, en ausencia de actividad bacteriostática o bactericida del DMSO al 40%, la actividad antimicrobiana observada puede atribuirse al extracto. Este hallazgo concuerda con otros informes que sugieren que los extractos de hojas de *B. orellana*, obtenidos por técnicas basadas en metanol o etanol, poseen actividad antimicrobiana contra cepas de referencia ATCC y aislados clínicos ^19^ ^,^ ^29^ ^,^ ^30^. Por lo tanto, sobre la base de nuestros resultados y los resultados reportados en otros estudios ^19^ ^,^ ^28^ ^-^ ^30^, es razonable considerar que el extracto evaluado posee actividad antimicrobiana.

La VB es una disbiosis caracterizada por cambios drásticos en la biota del tracto vaginal que dan lugar a una sustitución de la flora vaginal (predominantemente compuesta de lactobacilos) por bacterias anaerobias ^3^ ^,^ ^6^. *G. vaginalis* es una de las bacterias anaerobias más frecuentes causantes de VB, la cual puede formar, simbióticamente, una biopelícula polimicrobiana con bacterias anaerobias asociadas a VB, como *A. vaginae* y *Prevotella* spp ^2^ ^,^ ^6^ ^,^ ^31^. Otras bacterias anaerobias detectadas habitualmente en mujeres con VB son *Peptoestreptococcus* y *Mobiluncus* ^6^ ^,^ ^32^ ^,^ ^33^. Con respecto a los bajos valores observados de CMI y CBM, puede deducirse que el extracto evaluado en este estudio tiene una elevada actividad antimicrobiana frente a la mayoría de las cepas de referencia ATCC utilizadas y también frente a aislados clínicos de *G. vaginalis*. Sin embargo, es importante señalar que *P. bivia* y *V. parvula*, ambas cepas ATCC, mostraron baja susceptibilidad al extracto. Hasta donde sabemos, la actividad *in vitro* del extracto de *B. orellana* contra *G. vaginalis* y otros patógenos anaerobios apenas se ha descrito. Por lo tanto, se necesitan más estudios para validar nuestros hallazgos en una amplia variedad de aislados clínicos.

Los lactobacilos vaginales regulan el pH y protegen la mucosa frente al establecimiento de microorganismos patógenos ^3^ ^,^ ^33^. En este estudio, se utilizaron *L. crispatus* ATCC 33197 y aislados clínicos de *Lactobacillus* spp. como referencias del complejo de *Lactobacillus* que se encuentra en la vagina humana sana ^6^ ^,^ ^32^. Curiosamente, el extracto inhibió el crecimiento de los lactobacilos a concentraciones de 32,0 mg/mL. Sin embargo, nuestros resultados difieren de los descritos en otros estudios. Galindo-Cuspinera *et al*. ^34^ sugirieron que el extracto obtenido de frutos y semillas de *B. orellana* presentaba una actividad antimicrobiana limitada frente a *L. lactis* ATCC 11454 y *L. casei* ATCC 39539, y ninguna actividad frente a *L. plantarum* ATCC 700210. Del mismo modo, Ogunshe *et al*. ^35^ sugirieron que *B. orellana* tenía una actividad *in vitro* limitada contra varias cepas vaginales de *Lactobacillus* spp. En general, estos resultados discrepantes podrían explicarse por: i) el uso de diferentes métodos de extracción (es decir, tipo de extracto y disolvente utilizado) que dan lugar a una concentración variable de fitoquímicos, ii) el uso de diferentes partes de *B. orellana* (es decir, hojas, semillas, vainas o frutos) para la preparación del extracto, iii) la ubicación geográfica y la exposición a factores climáticos y medioambientales que afectan a *B. orellana*, y iv) por diferencias intrínsecas entre las cepas de *Lactobacillus* spp. utilizadas en otros estudios ^19^.

En general, nuestros hallazgos concuerdan con los de otros estudios que sugieren que el extracto de *B. orellana *posee actividad antimicrobiana ^17^ ^-^ ^19^ ^,^ ^28^ ^,^ ^30^. Un punto fuerte importante de este estudio *in vitro* es la evaluación de la actividad antimicrobiana del extracto frente a bacterias anaerobias representativas causantes de la VB y frente a bacterias representativas de la biota vaginal sana. Nuestros hallazgos pueden apoyar la ampliación y la innovación de la investigación para el desarrollo de posibles opciones terapéuticas. Sin embargo, también es importante tener en cuenta las limitaciones de este estudio *in vitro*. Hay múltiples fitoquímicos presentes en *B. orellana*, incluyendo flavonoides, polifenoles, taninos, quinonas, terpenoides y alcaloides que a través de procesos complejos ejercen actividad antimicrobiana ^19^. En este estudio preliminar no hemos caracterizado los compuestos fitoquímicos ni sus concentraciones; por lo tanto, no hemos podido establecer ni aislar la actividad antimicrobiana individual de cada componente del extracto frente a los aislados bacterianos estudiados. Además, es razonable suponer que la actividad antimicrobiana descrita no pueda extrapolarse a todas las especies de *B. orellana*, dadas las posibles diferencias intrínsecas entre ellas y las diferencias entre las formas de cultivo.

En resumen, nuestros experimentos *in vitro* sugieren que el extracto de *B. orellana* podría ser una fuente de compuestos antimicrobianos con una elevada actividad antimicrobiana selectiva contra la mayoría de las bacterias anaerobias asociadas a la VB, y con una actividad reducida contra las bacterias protectoras que se encuentran en la mucosa vaginal sana. Se necesitan más investigaciones para identificar el compuesto o los compuestos específicos con actividad antimicrobiana, para luego validar la actividad diferencial preliminarmente descrita aquí utilizando un diseño de estudio más riguroso y complejo.
